# Enhanced recovery after surgery program in Gynaecologic Oncological surgery in a minimally invasive techniques expert center

**DOI:** 10.1186/s12893-017-0332-9

**Published:** 2017-12-28

**Authors:** Eric Lambaudie, Alexandre de Nonneville, Clément Brun, Charlotte Laplane, Lam N’Guyen Duong, Jean-Marie Boher, Camille Jauffret, Guillaume Blache, Sophie Knight, Eric Cini, Gilles Houvenaeghel, Jean-Louis Blache

**Affiliations:** 1 Département de Chirurgie Oncologique 2, Institut Paoli Calmettes et CRCM, 232 Bd. Sainte-Marguerite, 13009 Marseille, France; 2 Aix-Marseille Univ, CNRS, INSERM, Institut Paoli-Calmettes, Department of Medical Oncology, CRCM, Marseille, France; 3 Département d’Anesthésie Réanimation, Institut Paoli Calmettes et CRCM, Marseille, France; 4 Aix-Marseille Univ, INSERM IRD, SESSTIM, Institut Paoli-Calmettes, Department of Clinical Research and Investigation, Biostatistics and Methodology Unit, Marseille, France

**Keywords:** Enhanced recovery after surgery, Fast-track programs, Gynaecological oncology surgery, Medical care enhancement, Minimally invasive techniques

## Abstract

**Background:**

Enhanced Recovery After Surgery Programs (ERP) includes multimodal approaches of perioperative patient’s clinical pathways designed to achieve early recovery after surgery and a decreased length of hospital stay (LOS).

**Methods:**

This observational study evaluated the implementation of ERP in gynaecologic oncological surgery in a minimally invasive techniques (MIT) expert center with more than 85% of procedures done with MIT. We compared a prospective cohort of 100 patients involved in ERP between December 2015 and June 2016 to a 100 patients control group, without ERP, previously managed in the same center between April 2015 and November 2015. All the included patients were referred for hysterectomy and/or pelvic or para-aortic lymphadenectomy for gynaecological cancer. The primary objective was to achieve a significant decrease of median LOS in the ERP group. Secondary objectives were decreases in proportion of patients achieving target LOS (2 days), morbidity and readmissions.

**Results:**

Except a disparity in oncological indications with a higher proportion of endometrial cancer in the group with ERP vs. the group without ERP (42% vs. 22%; *p = 0.003*), there were no differences in patient’s characteristics and surgical procedures. ERP were associated with decreases of median LOS (2.5 [0 to 11] days vs. 3 [1 to 14] days; *p = 0.002*) and proportion of discharged patient at target LOS (45% vs. 24%; *p = 0.002*). Morbidities occurred in 25% and 26% in the groups with and without ERP and readmission rates were respectively of 6% and 8%, without any significant difference.

**Conclusion:**

ERP in gynaecologic oncological surgery is associated with a decrease of LOS without increases of morbidity or readmission rates, even in a center with a high proportion of MIT. Although it is already widely accepted that MIT improves early recovery, our study shows that the addition of ERP’s clinical pathways improve surgical outcomes and patient care management.

**Electronic supplementary material:**

The online version of this article (10.1186/s12893-017-0332-9) contains supplementary material, which is available to authorized users.

## Background

Enhanced Recovery Programs (ERP), or “fast-track” programs, are a multimodal approach of perioperative patient’s clinical pathways designed to achieve early recovery after surgery and decrease length of hospital stay (LOS). This patient-centered management was defined in the 1990’s by Kehlet et al. [1, 2], and refers to a specific organization in which an informed patient plays an active role. The key elements of ERP include preoperative counsel, optimization of nutrition, standardized analgesic and anaesthetic regimens and early mobilization. Although ERP have become an important focus of perioperative management after many type of surgeries [3], initial studies in gynaecology surgery only included hysterectomies for benign indications [4–6] or precancerous lesions [7, 8]. Only few studies have evaluated ERP role in gynaecological oncology [9–11], all with the same conclusion of achieving shorter LOS and enhancing recovery. Furthermore, most publications are limited to one disease or one type of procedure, and didn’t report the implementation of ERP in a established high proportion of minimally invasive techniques (MIT) context. Our institution introduced ERP in 2015 for gynaecological cancer patients (cervical, endometrial and ovarian cancers) admitted for MIT as for open surgery. MIT, which constitutes by itself one of the most important component of ERP’s clinical pathways [12], has been shown to be safe and feasible with the same oncological results than open procedures with better cosmetic results and obviously, less postoperative pain, and shorter hospital stays [13].

This study evaluated the implementation of ERP in gynaecologic oncological surgery in a MIT expert center with more than 85% of procedures done with MIT. The primary objective was to achieve a significant decrease of median LOS in the ERP group. Secondary objectives were decreases of prevalence of postoperative complications and readmissions, and increase of patients achieving a target LOS of 2 days.

## Methods

### Study design and data source

This prospective observational study was conducted at the Institut Paoli-Calmettes which is one of the 18 French comprehensive cancer centers, located in Marseille, FRANCE, between December 2015 and June 2016. Consecutive patients undergoing hysterectomy and/or pelvic or para-aortic lymphadenectomy for gynaecological cancer (cervical, endometrial, ovarian cancer without bowel resection or other including borderline ovarian tumor, endometrial hyperplasia and cervical intraepithelial neoplasia) were identified. The only exclusion criterion was post-operative admission to intensive care unit (ICU) for more than one night.

All staff involved, including surgeons, anaesthesiologists, nurses and dieticians, received specific training regarding three defined phases of Enhanced Recovery Pathway management (Table 1), including latest published recommendations [14–18]. Written procedures were established and spread to all the actors of the clinical pathway. Further description of Paoli-Calmettes Institute’s ERP is available in Annex 1 (Additional file 1). Since association between improved protocol adherence and improved postoperative outcomes is described [19, 20], we evaluated compliance criteria which are summarized in Table 2. Good compliance was defined as ≥70% score per criteria. After hospital discharge, follow-up was conducted at home to day 7 through a network of nurses, in order to detect early perioperative complications and to evaluate the compliance to the ERP and post-operative instructions. In addition, the post-operative nurse coordinator organized telephone interviews at days 1, 7 and 30, to record all occurrences of readmission in other hospitals and/or long-term post-operative complications. Per and post-operative complications were collected according to the Clavien-Dindo classification [21].

**Table 1:** Enhanced Recovery Pathway

I. Preoperative	
Diet	• Evening before surgery: may eat until midnight • Clear fluids up to 2 h before procedure, including 50 g Carbohydrate in 400 ml (Nutricia ®)
Bowel preparation	• No systematic use of mechanical bowel preparation; rectal enemas still performed
Preoperative sedation	• No systematic preoperative sedation, unless anxious crisis
II. Intraoperative	
Nausea and vomiting prophylaxis	• Before incision: Dexamethasone 8 mg IV once (4 mg if age > 80 or weight < 50 kg) • Before incision closure: ✓ Droperidol 1.25 mg IV once ✓ Ondansetron 4 mg IV once in high risk patient (Apfel score > 3)
Fluid balance	• Goal: maintain intraoperative Zero Fluid Balance ✓ crystalloid maintenance administration: 3 ml/kg/h for laparoscopy; 5 ml/kg/h for open ✓ In case of blood loss: replacement according to institutional protocol
Analgesia	• Continuous AIVOC Remifentanyl at discretion of anesthesiologist, supplemented with IV Ketamine (0.5 mg/kg at induction and 0,15 mg/kg hourly boluses) • IV Lidocaine 1 mg/kg at induction, then 1,5 mg/kg/h until the end of surgery • Before the end of surgery: IV 1000 mg Acetaminophen, 100 mg Ketoprofen (if no contraindication) and 20 mg Nefopam • In case of laparoscopy: injection of Naropein (2 mg/kg maximum) at incision site • In case of laparotomy: bilateral single shot Tap Block with Naropein (2 mg/kg maximum). No epidural analgesia
III. Postoperative	
Activity	• Evening of POD 0: out of bed more than 2 h, including sitting in chair • POD 1 and until discharge: out of bed more than 4 h, including deambulation in ward and sitting in chair • Patient up in chair for all meals • Removal of urinary catheter by POD 1
Diet	• No nasogastric tube; if nasogastric tube used intraoperatively, removal at extubation • Patient encouraged to start clear fluid 2 h after procedure • POD 0: Patient encouraged to start free diet. In case of difficulties, one to two boxes of liquid nutritional supplement • POD 1 until discharge: free diet. Encourage daily oral fluid intake (1500–2500 mL)
Analgesia	• Goal: opioid sparing; no IV morphin patient-controlled analgesia • Scheduled oral level II opioids ✓ Izalgi® (Acetominophen 500 mg + Opium powder) orally every 6 h • Scheduled Acetaminophen ✓ Acetaminophen 500 mg orally every 6 h ✓ For patients with no hepatic disease: Maximum Acetaminophen should not exceed 4000 mg/24 h from all sources including Izalgi® • Scheduled NSAIDs if no contraindication: renal impairment with creatinine clearance less than 40 ml/min or hepatic disease ✓ Ketoprofen 100 mg orally twice daily (start no sooner than 6 h after the intraoperative dose), until POD 2 ✓ If patient unable to take NSAIDs: Tramadol 50 mg orally every 6 h. • Breakthrough pain ✓ Oxycodone 5–10 mg orally every 4 h if needed
Fluid balance	• Peripheral IV catheter locked on departure from PACU • In case of laparotomy: Fluid maintenance at 40 mL/h until 8:00 am the day after surgery and then discontinued

*Abbreviations*: *IV* intra venous, *PACU* Post anaesthesia care unit, *POD* post operative day

**Table 2:** Perioperative care ERP interventions and definition of adherence

Preoperative	
Preadmission education	Patient received preoperative counselling from a nurse and a physician, and a dedicated booklet including information on recovery goals and expectation about hospital stay.
Selective MBP	No MBP used for resection.
Carbohydrate loading	Preoperative carbohydrate intake until 2 h before anaesthesia (50 g carbohydrate in 400 mL fluid).
No long-acting sedation	No long-acting sedating medication used before surgery (e.g., opioids, antihistamines, benzodiazepines).
Intraoperative	
Antibiotic prophylaxis	Antibiotic prophylaxis completed prior to surgical incision
IV Lidocaine	Continuous infusion: 1.5 mg/kg/h from the beginning to the end of surgery.
Laparoscopic approach	Successfully completed laparoscopic resection.
Zero Fluid Balanced	Intraoperative maintenance fluids, excluding replacement of blood loss: for laparoscopy: 3 ml/kg/h; for open surgery: 5 ml/kg/h.
PONV	Multimodal prophylaxis administered, with at least to drugs including Dexamethasone.
No abdominal or pelvic drainage	No resection-site drainage used.
Normothermia	Body temperature measured at the end of surgery >36.0 _C.
Preventive Opioid-sparing Multimodal	Acetaminophen, NSAIDs (unless complication), Nefopam: IV first dose administered intraoperatively.
Analgesia	Loco-regional analgesia performed (injection at incision site or bilateral TAP block).
Postoperative	
Opioid-sparing multimodal analgesia	Use of opioid-sparing strategies including, abdominal trunk block and oral analgesia: acetaminophen, NSAIDs (unless complication), Level II opioids.
Free diet on POD 0	Patient received one meal with regular food by POD 0.
Early mobilization out of bed on POD0	Patient mobilized out of bed after surgery by POD 0.
Early ablation of IV fluid infusion	Ablation of intravenous fluid infusion by POD 0.
Early removal of urinary drainage	Removal of urinary catheter by POD 1.
TED prophylaxis	TED prophylaxis with low molecular weight heparin.
Avoidance of nasogastric tube	Nasogastric tube removed at the end of general anaesthesia.

*Abbreviations*: *MBP* mechanical bowel preparation, *PONV* prevention of nausea and vomiting

To confirm the interest of ERP in terms of hospital’s length of stay (nights spent at hospital, including the night before surgery) and morbidity (including readmission), we compared our prospective cohort to a 100 patients control group, without ERP, matched on Age, American Society of Anesthesiologists (ASA) score and type of procedure (conventional laparoscopy or robotically assisted laparoscopy or open surgery) from a historical cohort, previously managed in the same center between April 2015 and November 2015.

All procedures performed in this study involving human participants were done in accordance with the French ethical standards and with the 2008 Helsinki declaration. All included patients provided written informed consent before surgery. This work was approved by our institutional review board (IPC Comité d’Orientation Stratégique).

### Statistical analysis

Categorical variables were described using counts and frequencies, and quantitative variables were described using mean, medians and ranges. Patients’ characteristics and distribution were compared with Mann-Whitney U and χ2 tests. The level of statistical significance was set at α = 0.05. Statistical analyses were carried out with the SAS® software version 9.3. We followed the reporting recommendations specified in the STROBE (Strengthening the Reporting of Observational Studies in Epidemiology) Statement [22].

## Results

### ERP compliance

**Fig. 1:**
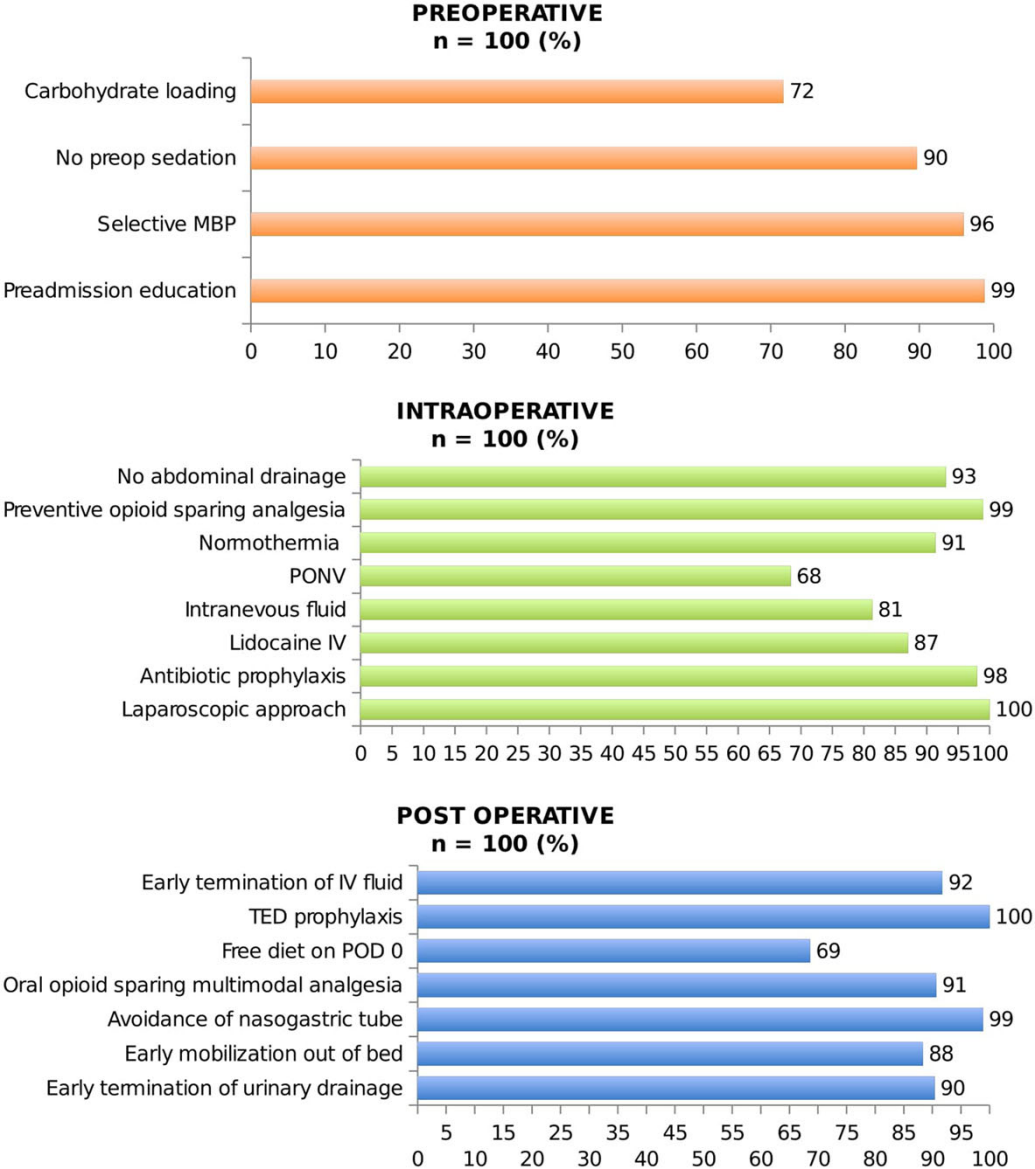
Compliance (%) with peri operative ERP’s criteria. Abbreviations: MBP, mechanical bowel preparation; PONV, prevention of nausea and vomiting; TED, thromboembolic deterrent

Compliances with perioperative ERP’s criteria are reported in Fig. 1. Overall compliance rate was 90%. Each criteria compliance varies from 68 to 100%. During the post-operative period, only one patient required to maintain nasogastric intubation and 12% of patients had abdominal drainages that were removed on day 2 according to our recommendations. At day 1, urinary catheter was removed in 94% patients, 97% had returned to a regular diet, 87% patients were able to walk in the corridors and 92% had undergone early mobilization. Only 10% suffered from immediate post-operative nausea. Post-operative level of haemoglobin was >10 g/dL for 76% patients and thromboembolism prophylaxis has been administrated in 100% of cases.

### Characteristics of the study population and surgical procedures

Patients’ characteristics before and after ERP’s implementation are summarized in Table 3. No difference was observed between matching criteria: Mean age, ASA score and surgical procedures were similar between the 2 groups. We observed a significant difference concerning oncological indications in relation with a higher proportion of endometrial cancer in the ERP group (42% vs. 22% before ERP; *p = 0.003*). The others indications were distributed as follow: cervical cancer 39% vs. 42%, ovarian cancer 14% vs. 22% and others 5% vs. 14%, without significant difference. Most of the procedures were performed by conventional or robotic assisted laparoscopy (87% after ERP vs. 88% before ERP) and only few of the procedures were performed by open surgery (13% after ERP vs. 12% before ERP). All the procedures are detailed in Table 3, hysterectomy and bilateral oophorectomy, hysterectomy and bilateral oophorectomy with lymphadenectomy and isolated lymphadenectomy, without any significant difference before and after ERP implementation. Conversion rate from laparoscopy to open was similar in the 2 groups (2%).

**Table 3:** Patient’s characteristics and surgical procedures before and after ERP implementation

	Before ERP(*n* = 100)	After ERP(*n* = 100)	*p*-value
Age (years)			
Mean (+/− SD)	59,01 (+/− 12,35)	59,37 (+/− 13,13)	NS
Median (Min/Max)	60 (16 / 87)	60 (27 / 91)	
ASA score:			
ASA 1 (%)	21	24	NS
ASA 2 (%)	69	65	
ASA 3 (%)	10	11	
BMI (kg/m2)			
Mean (+/− SD)	24,2 (+/− 4,98)	27,3 (+/− 7,89)	0.001
Median (Min/Max)	23 (16 / 41)	25 (18 / 63)	
Oncological indications (%)			
Endometrial cancer*	22	42	0.003*
Cervical cancer	42	39	
Ovarian cancer	22	14	
Other (Border line ovarian tumor, endometrial hyperplasia, CIN)	14	5	
Surgical approaches			
Conventional lap / Robotic assisted lap	88	87	NS
Open	12	13	
Conversion to open	2	2	
Surgical procedures			NS
Total Hysterectomy**	43	48	
Total Hysterectomy** with pelvic lymphadenectomy	11	16	NS
Total Hysterectomy** with pelvic and aortic lymphadenectomy	9	9	
Isolated Lymphadenectomy:	37	27	
Pelvic lymphadenectomy	12	4	
Para-aortic lymphadenectomy	21	18	
Both	4	5	

*Abbreviations: BMI* body mass index, *SD* standard deviation, *CIN* Cervical Intraepithelial Neoplasia

*significative difference in oncological indications in relation with the rate of endometrial cancer, higher in the group after ERP

**total hysterectomy was always associated with bilateral oophorectomy

### Length of stay

**Fig. 2:**
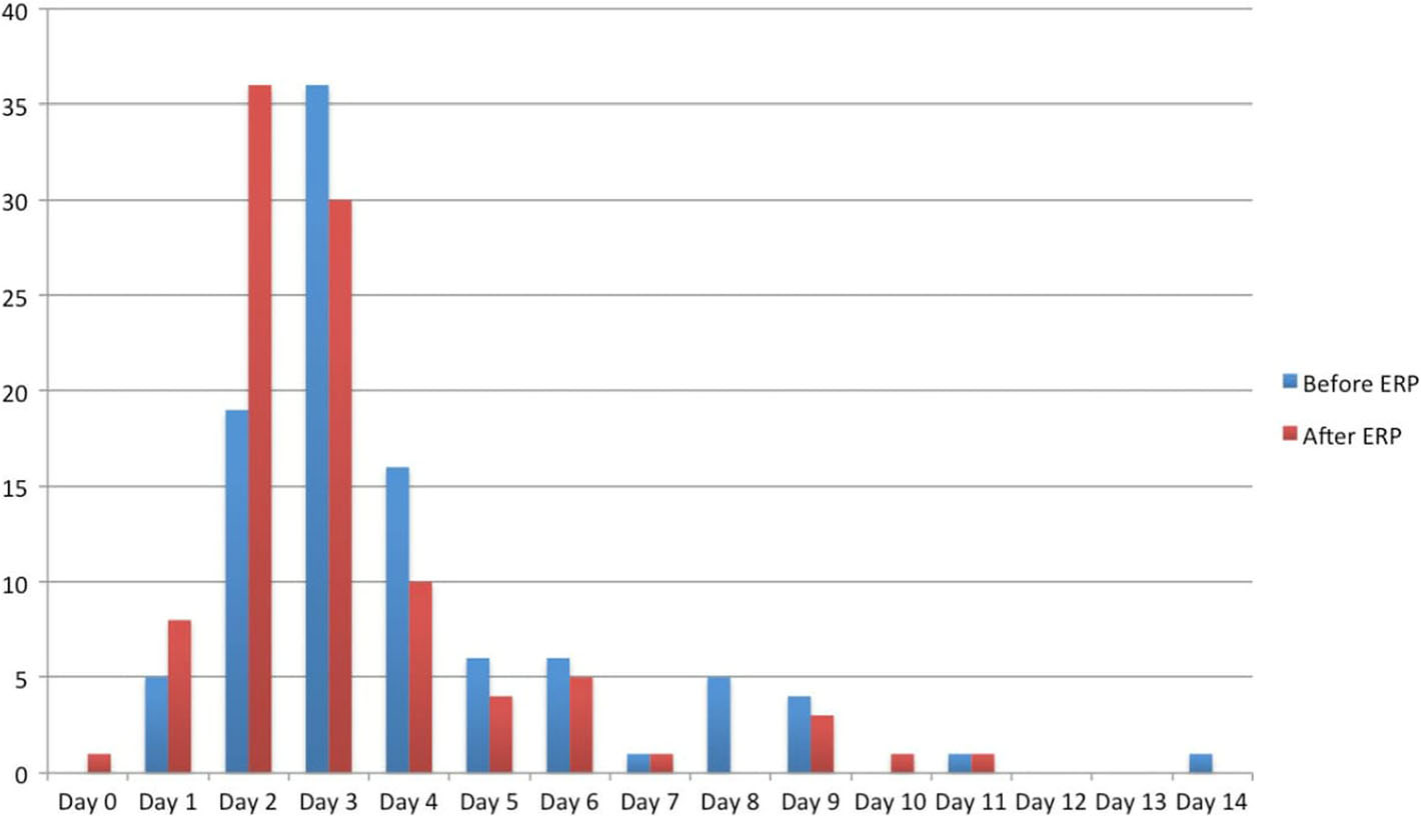
Length of stay (in nights spent) before and after ERP

ERP were associated with decreases of median LOS (2.5 days [0 to 11] vs. 3 days [1 to 14]; *p = 0.002*) and proportion of discharged patient at target LOS of 2 days (45% vs. 24%; *p = 0.002*) (Table 4). The analysis by surgical approaches reveals a decrease of median LOS for laparoscopic approaches (2 [1 to 9] days vs. 3 [1 to 9] days; *p = 0.0005*) but only a tendency for open surgical procedures (6 [3 to 11] days vs. 8 [4 to 14] days; *p = 0.17*). Regarding MIT, the ratio of pre- and post ERP median LOS was even better in robotically assisted laparoscopy than in conventional laparoscopy 0.76 vs. 0.85 (Table 4). Lengths of stay (in nights spent) before and after ERP are summarized in Fig. 2.

**Table 4:** Analysis of hospital stay before and after implementation of ERP

	Before ERP(*n* = 100)	After ERP(*n* = 100)	*p*-value
Primary Hospital stay			
Mean (+/− SD)	3.89 (+/− 2.3)	3.15 (+/− 1.97)	0.002
Median (Min/Max)	3 (1/ 14)	2.5 (0 / 11)	
Hospital stay / Surgical approaches			
Minimally Invasive Techniques	*n* = 88	*n* = 87	
Mean (+/− SD)	3.33 (+/− 1.58)	2.67 (+/− 1.29)	0.003
Median (Min/Max)	3 (1 / 9)	2 (1 / 9)	
Conventional laparoscopy	*n* = 53	*n* = 53	
Mean (+/− SD)	2.86 (+/− 0.97)	2.42 (+/− 0.95)	0.022
Median (Min/Max)	3 (1 / 6)	2 (1 / 6)	
Robotically assisted laparoscopy	*n* = 35	*n* = 34	
Mean (+/− SD)	4.1 (+/− 2.01)	3.1 (+/− 1.6)	0.028
Median (Min/Max)	4 (1 / 9)	3 (0 / 9)	
Open surgery	*n* = 12	*n* = 13	
Mean (+/− SD)	8 (+/− 2.63)	6.38 (+/− 2.6)	0.14
Median (Min/Max)	8 (4 / 14)	6 (3 / 11)	
Hospital Stay			
≤ 2 days	24	45	0.002
> 2 days	76	55

### Postoperative complications and readmissions

Overall complication rate was 25% in the ERP group vs. 26% in the group without ERP up to 30 days after discharge, including per and post operative complications (Table 5). Considering only grade III-IV complications, we observed 3 grade III post-operative complications in both groups. No grade IV occurred. The readmission rate in relation with post-operative complications up to 30 days after surgery was 6% in the ERP group and 4% in the group without ERP. An extended description of complications that have occurred only in patients who have undergone minimally invasive techniques is available in Additional file 2: Table S1.

**Table 5:** Analysis of morbidity before and after implementation of ERP

	Before ERP(*n* = 100)	After ERP(*n* = 100)	*p*-value
Total	26	25	NS
Per operative complications	1	2	NS
	Bladder injury (*n* = 1)	Bladder injury (*n* = 1)	
		Obturator nerve section (*n* = 1)	
Early post operative complications*(during hospitalization)	11	8	NS
Grade I/II	10	8	
	Urinary Infection (*n* = 3)	Urinary Infection (*n* = 2)	
	Ileus (*n* = 1)	Vaginal Bleeding (*n* = 2)	
	Vaginal Bleeding (*n* = 3)	Lymphorrhea (*n* = 1)	
	Lymphorrhea (*n* = 1)	Obturator nerve injury (*n* = 1)	
	Dysuria (*n* = 2)	Dysuria (*n* = 2)	
Grade III/IV	1	0	
	Abdominal wall hematoma IIIB (*n* = 1)		
Post operative complications*(after discharge up to 30 days)	14	15	NS
Grade I/II	12	12	
	Lymphocele (*n* = 6)	Urinary Infection (*n* = 2)**	
	Dysuria (*n* = 2)	Vaginal cuff leakage (*n* = 1)**	
	Urinary infection (*n* = 1)**	Vaginal bleeding (*n* = 4)	
	Nephritic colitis (*n* = 1)**	Lymphocele (*n* = 4)	
	Muscular and skeletic pain (*n* = 2)	Dysesthesia (*n* = 1)	
Grade III/IV	2	3	
	Chylous ascites IIIA (*n* = 1)**	Symptomatic lymphocele IIIA (*n* = 1)**	
	Symptomatic lymphocele IIIA (*n* = 1)**	Lymphocele surinfected IIIB (*n* = 1)**	
		Deep hematoma IIIB (*n* = 1)**	
Readmissions in relation with post operative complications**	4	6	NS

*According to Clavien Dindo Classification

**readmissions in relation with complications described in "post operative complications after discharge up to 30 days"

## Discussion

The present study demonstrates that ERP implementation in gynaecologic oncological surgery is associated with LOS decrease without increases of morbidity or readmission rates, even in a high-volume cancer center where MIS is developed and routinely used since 15 years. Although ERP have become an important focus of perioperative management after many type of surgeries [3, 23], the recovery benefits following gynaecologic oncological surgery remain uncertain when MIT are already implanted. Recently, De Groot et al. published the design of a stepped strategy that aims at the nationwide implementation of the ERP in major gynaecological surgery in the Netherlands [24]. This ambitious trial is ongoing with the objective to evaluate effectiveness and costs of a stepped implementation approach that is characterized by tailoring the intensity of implementation activities to the needs of organizations and local barriers for change, in comparison with the generic breakthrough strategy that is usually applied in large-scale improvement projects. In accordance with De Groot’s hypothesis, the high proportion of MIT already established in our department could have participated to the success of our study. One of the most important difficulties observed to implement such a program is the need of collaboration between different health professionals and different specialties. ERP required a multidisciplinary team involving surgeons, anaesthesiologists and nurses to establish standardized clinical pathway, train and encourage the whole team to participate. ERP’s compliance must be evaluated during the implementation phase because the success of this program is directly linked with the percentage of adherence [19]. The selection of our ERP’s preoperative, peroperative and post-operative criteria’s has been made according to the current recommendations that are reproducible, acceptable for patients and adapted to our practices. In our series, overall compliance rate was at 90% (68 to 100% according the different criteria) and led to a significant reduction of LOS. There has been a recent widespread international ‘paradigm shift’ to new perioperative systems, based on a multidisciplinary team providing an integrated process of care. Lee et al. synthesize the evidence supporting these new systems moving to a more proactive, coordinated and team-based approach [25]. In addition, it seems that the adding of home visits to telephone interviews, as we provide in our ERP, are more effective than telephone interviews alone to prevent readmission [26]. In relation with these observations, it seems that a post-operative coordination is necessary to develop perioperative surgical at home in a safe way for our patients. There are multiple considerations in how to optimize ERP implementation. The most crucial at our institution was to invest in a dedicated nurse to oversee the process from hospitalization to home. In addition, to support this strategy we have organized an “at home” follow up with a network of care with nurses, from the day of discharge to the day 7 after discharge, to detect early perioperative complications, and to evaluate the compliance to the ERP and post-operative instructions. In a retrospective study, Shiavone et al. [27] evaluated 128,634 women who had undergone a laparoscopic hysterectomy and showed a 4.0% rate of re-evaluation within 60 days for women discharged on the same day of surgery, 3.6%, for those discharged after a 24-h stay, and 5.1% for those whose length of stay was 2 days or longer, respectively (*p < 0.001*). Moreover, patients discharged on post-operative day 1 were 11% less likely to require revaluation than women discharged on the day of surgery. Current constraints in France are due to the lack of availability of operating rooms and hospital beds; ERP appears to be a solution to improved healthcare system without impairing patients quality of life or satisfaction [8, 28, 29, 20]. The LAFA Study suggest that the success of patient’s centred clinical pathway is linked to the surgical approach [12]. MIT was the only independent predictive factor to reduce LOS in this study. We showed here that ERP has participated to a quiet small, but statistically significant, reduction of primary hospital stay, even with a high rate of MIS. Additionally, if the most important gain in term of LOS in subgroups analysis appears to be in open surgery, this reduction didn’t reach the significance level, probably due to the low number of patients. Complication rate was consistent with those reported in literature, in relation with surgical complications and particularly lymphatic dissections [30].

Despite careful methodology, our study has some limits, lying mainly in the small number of patients and in the retrospective analysis of the control group with potential selection bias and lack of standardization in the monitoring of post-operative complications. However, this last point would rather lead to an underestimation of the complications in the group without ERP and thus to increase the difference in favour of ERP implementation. Our study has also strengths as the matching of the two populations and the high rate of MIT procedures in both groups, which allowed us to conclude on ERP implementation in a center where the practices of MIT are already extended.

## Conclusions

Although it is already widely accepted that MIT improves early recovery, our study shows that the addition of ERP’s clinical pathways improves surgical outcomes and patient care management. We observed a decrease of LOS in gynaecologic oncological surgery indications, without increases of morbidity or readmission rates.

## Additional files

Additional file 1: Annex 1. Description of Paoli-Calmettes Institute’s ERP. (DOCX 22 kb)

Additional file 2: Table S1. Analysis of morbidity before and after implementation of ERP in patients who have undergone minimally invasive techniques. (DOCX 94 kb)

## Abbreviations

ASA: American Society of Anesthesiologists
BMI: Body mass index
CIN: Cervical Intraepithelial Neoplasia
ERP: Enhanced Recovery After Surgery Programs
ICU: Intensive Care Unit
LOS: Length Of hospital Stay
MIT: Minimally Invasive Techniques
SD: Standard deviation
