# Supplementary material for: Enhanced recovery after surgery program in Gynaecologic Oncological surgery in a minimally invasive techniques expert center
Source: BMC Surg. 2017 Dec 28;17:136. doi: 10.1186/s12893-017-0332-9 (PMC5745717; doi:10.1186/s12893-017-0332-9)
Supplement: Supplementary file 1 — Description of Paoli-Calmettes Institute’s ERP. (DOCX 22 kb) [file 12893_2017_332_MOESM1_ESM.docx]

**Annex 1. Description of Paoli-Calmettes Institute’s ERP:**

***Preoperative period***

This step included the surgical consultation and anaesthetic evaluation. Patients received verbal and written ERP information by a clinic nurse. In case of ovarian cancer with neoadjuvant chemotherapy, immunonutrition (Oral Impact®) was recommended 7 days before surgery. Use of tobacco and/or alcohol was assessed and a consultation with a de dedicated health professionals was systematically offered to concerned patients. If anemia or undiagnosed diabetes/hyperglycemic states were diagnosed during anaesthetic evaluation, interventions addressing these factors prior to elective surgery were conducted.

***Peroperative period***

The WHO surgical safety check-list was systematically performed and antibiotic prophylaxis administered. Hypothermia was prevented by an active warming device.

A multimodal preventive approach to prevention of nausea and vomiting (PONV) with 2 or 3 antiemetic agents (including Dexamethasone) was used before the end of surgery. Restrictive or liberal fluid administration was avoided with a zero balance fluid therapy objective. Goal Directed Fluid Therapy with oesophageal doppler probe was used in high risk patients. Opioid free multimodal analgesic regimen (Paracetamol, Ketoprofen, Nefopam) was administered preventively before the end of surgery. Abdominal drainage was not systematically performed particularly in case of laparoscopic approach.

***Postoperative period***

In post anaesthesia care unit (PACU), nasogastric tube, urinary and IV catheter were removed, in the absence of surgical or anaesthetic contraindication. Opioid free multimodal analgesic regimen was orally taken from POD 0 (Paracetamol, Ketoprofen). A morphine rescue treatment was possible with oral Oxycodone. Chewing-gum was systematically proposed to limit the risk of postoperative ileus.

Patients were encouraged to drink as soon as possible, 2 hours after the end of surgery (after PACU). Patients should be out of bed as early as possible at DO and a regular diet should be offered too. Patients should wear compression stockings and should have low molecular weight heparin (LMWH) prophylaxis for 4 weeks postoperatively.

Discharge criterions were standardized: Pain controlled by oral analgesics - No signs of infection (T°<38°c, GB <10 000, pulse < 120/min) - Feeding: solid food - Intestinal activity at least for flatus - Patient accepting discharge.

Our ERP protocol has anaesthesia feature differences compared with others:

We decided to omit epidural analgesia even in patients undergoing open surgery given concern about hypotension and inappropriate treatment with fluid boluses instead of vasopressors. Moreover, epidural analgesia may impair early mobilization: we choose to use during intraoperative period continuous IV lidocaine and low dose of ketamine boluses.
