# Supplementary material for: Enhanced recovery after surgery program in Gynaecologic Oncological surgery in a minimally invasive techniques expert center
Source: BMC Surg. 2017 Dec 28;17:136. doi: 10.1186/s12893-017-0332-9 (PMC5745717; doi:10.1186/s12893-017-0332-9)
Supplement: Supplementary file 2 — Analysis of morbidity before and after implementation of ERP in patients who have undergone minimally invasive techniques. (DOCX 94 kb) [file 12893_2017_332_MOESM2_ESM.docx]

**Supplementary table 1.** Analysis of morbidity before and after implementation of ERP in patients who have undergone minimally invasive techniques

|  | **Before ERP (n=100)** | | **After ERP (n=100)** | | **p-value** |
| --- | --- | --- | --- | --- | --- |
| **Type of laparoscopy** | **Conventional (n=53)** | **Robotically assisted (n=35)** | **Conventional (n=53)** | **Robotically assisted (n=34)** |  |
| **Total** | **11** | **8** | **12** | **7** | NS |
| **Per operative complications** | 1 Bladder injury (n=1) | 0 | 1 Obturator nerve section (n=1) | 0 | NS |
| **Early post operative complications*** | 3 | 3 | 2 | 2 | NS |
| Grade I/II | 2 | 3 | 2 | 2 |  |
|  | Dysuria (n=1) | Dysuria (n=2) | Vaginal Bleeding (n=1) | Dysuria (n=1) |  |
|  | Lymphorrhea (n=1) | Vaginal Bleeding (n=1) | Obturator nerve injury (n=1) | Urinary Infection (n=1) |  |
| Grade III/IV | 1 | 0 | 0 | 0 |  |
|  | Abdominal wall hematoma IIIB (n=1) |  |  |  |  |
| **Post operative complications*** | 7 | 5 | 9 | 5 | NS |
| Grade I/II | 6 | 4 | 6 | 5 |  |
|  | Lymphocele (n=3) | Lymphocele (n=2) | Vaginal Bleeding (n=2) | Vaginal Bleeding (n=2) |  |
|  | Nephritic colitis (n=1) | Muscular and skeletic pain (n=1) | Urinary infection (n=1) | Lymphocele (n=2) |  |
|  | Dysuria (n=1) | Dysuria (n=1) | Vaginal cuff leakage (n=1) | Urinary infection (n=1) |  |
|  | Muscular and skeletic pain (n=1) |  | Lymphocele (n=1) |  |  |
|  |  |  | Dysesthesia (n=1) |  |  |
| Grade III/IV | 1 | 1 | 3 | 0 |  |
|  | Symptomatic lymphocele IIIA (n=1) | Chylous ascites IIIA (n=1) | Symptomatic lymphocele IIIA (n=1) |  |  |
|  |  |  | Lymphocele surinfected IIIB (n=1) |  |  |
|  |  |  | Deep hematoma IIIB (n=1) |  |  |
| **According to Clavien Dindo Classification* | |  |  |  |  |
